# Mismatch-Assisted Toehold Exchange Cascades for Magnetic Nanoparticle-based Nucleic Acid Diagnostics

**DOI:** 10.1021/jacsau.5c00985

**Published:** 2025-09-04

**Authors:** Rebecca Sack, Joshua Evans, Florian Wolgast, Meinhard Schilling, Thilo Viereck, Petr Šulc, Aidin Lak

**Affiliations:** † Institute for Electrical Measurement Science and Fundamental Electrical Engineering and Laboratory for Emerging Nanometrology (LENA), Hans-Sommer-Str. 66, Braunschweig 38106, Germany; ‡ School of Molecular Sciences and Center for Molecular Design and Biomimetics, The Biodesign Institute, 7864 Arizona State University, 1001 South McAllister Avenue, Tempe, Arizona 85281, United States; § School of Natural Sciences, Department of Bioscience, Technical University Munich, Garching 85748, Germany

**Keywords:** magnetic nanoparticles, nanoparticle-based DNA cascades, mismatch, toehold exchange, kinetics, ac magnetic susceptibility, amplification-free detection, magnetic particle spectroscopy

## Abstract

Sensitive, simple, and rapid detection of nucleic acid sequences at point-of-care settings is still an unmet quest. Magnetic readout assays combined with toehold-mediated strand displacement-based circuits are amplification- and wash-free, essential features for contributing to this demand. Nevertheless, nonenzymatic strand displacement circuits are slow, with low sensitivity for early disease diagnostics. Here, we propose novel mismatch-assisted toehold exchange (MATE) magnetic cascades, wherein magnetic susceptibility increases by dissociation of magnetic nanoparticles (MNPs) from engineered magnetic clusters upon detection of a nucleic acid target in solution. The MATE relies on the generation of an allosteric toehold by spontaneous dissociation to efficiently recycle the target, amplify magnetic signal output, and enhance the assay’s kinetics. We show that introducing a mismatch in the allosteric toehold domain enhances the overall declustering kinetics 7-fold, as also confirmed with oxDNA simulations, with the largest effect gained for the mismatch being closest to where the branch migration by the target ends. By integrating MATE into magnetic diagnostics cascades, we demonstrate similar sensitivity in a 12-fold shorter assay time compared to our previous circuit design. Our work makes a major leap toward bringing MNP-based diagnostics much closer to the clinical point-of-care settings by offering a simple, rapid, isothermal, and nonenzymatic assay workflow.

## Graphical abstract

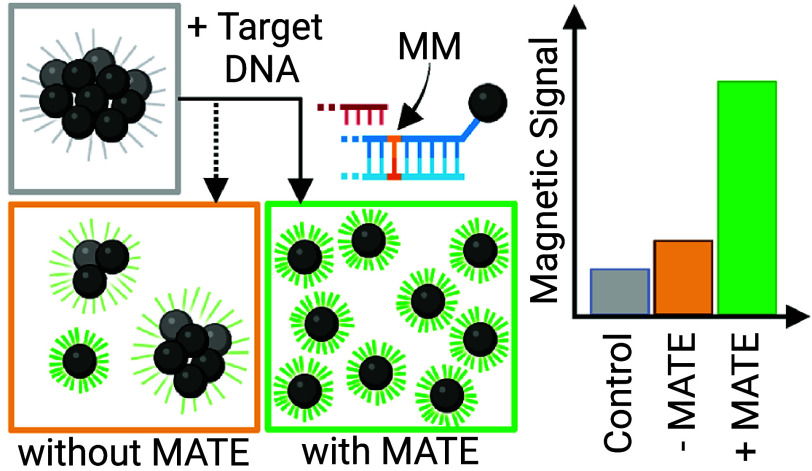

## Introduction

Certain nucleic acid sequences are highly specific to bacterial^1,2^ and viral^3–6^ genomes, as well as cancer,^7–10^ and are thus the main target for early and accurate disease diagnostics. Reverse transcription polymerase chain reaction (RT-PCR), the “gold standard” of molecular assays, can detect a few nucleic acid copies through enzymatic amplification reactions.^11,12^ However, PCR requires expensive equipment and reagents and is time-consuming and prone to contamination, making its application in point-of-care (POC) settings impossible. Particularly during the COVID-19 pandemic, amplification-free biosensing concepts, including CRISPR-based diagnostics,^13–15^ nanopore readout,^16,17^ DNA origami,^18,19^ and DNA circuits, have been further developed. DNA circuits based on toehold-mediated strand displacement (TMSD) are among the most studied circuits, owing to their versatility, programmability, and amplification-free operation at ambient conditions.^20–24^ While TMSD-based circuits simplify experimental requirements greatly, as they are nonenzymatic and isothermal,^25–27^ they lead to weak signal gain at low target concentrations and have slow kinetics, by TMSD being a statistical process of toehold formation and branch migration.^21^

Over the past few years, the kinetics of TMSD circuits have been enhanced by using enzymes and proteins.^28–31^ However, for real-world applications and especially for testing in low-income countries, the low-temperature transport and storage of enzymes and expensive reagents are not an option. To realize nonenzymatic circuits with fast kinetics, elaborate amplification circuits have been employed. Numerous DNA circuits such as catalytic hairpin assembly,^32,33^ hybridization chain reactions,^34,35^ and circuit reactions^25,36^ were designed to amplify the signal gained per target. Circuit reactions can utilize toehold exchange, which involves partial displacement and spontaneous dissociation of an incumbent strand, opening an allosteric toehold for a so-called fuel sequence, which recycles the target and enhances the reaction kinetics.^36,37^ A study on toehold exchange processes has demonstrated the positive effect of the primary toehold being one base-pair longer than the allosteric toehold on the reaction kinetics.^37^ Other studies have demonstrated the significance of sequence^38,39^ and complementarity of utilized DNA strands, as mismatched base-pairs accelerate or decrease the kinetics of TMSD-based circuits substantially.^40–44^ Implementing a mismatch in the allosteric toehold domain can lead to a synergic effect and significantly enhance the kinetics of TMSD-based circuits. However, it has not yet been explored how the position of said mismatch impacts the kinetics of nonenzymatic diagnostic cascades.

TMSD-based circuits often use colorimetric or fluorescent readout methods and rely on reporter duplexes to monitor their kinetics.^24,37,41^ Alternative readout approaches utilize functionalized gold^45–48^ or MNPs^8,49–56^ as markers. While fluorescence-based assays are affected by background molecules,^57,58^ magnetic measurements are potentially not influenced by cell debris, background macromolecules, and proteins present in complex biological samples, as they are nonmagnetic.^59,60^ Moreover, magnetic fields are negligibly attenuated in opaque media, thus allowing the detection of nucleic acids directly on unprocessed samples, unlike methods based on visible light. The dynamic magnetic response of MNPs to moderate alternating magnetic fields changes in a highly specific manner upon molecular binding between receptors on MNPs and targets in solution.^26,47,54,61–63^ The change in particle hydrodynamic size, resulting from target recognition, is picked up within a minute with magnetic particle spectroscopy (MPS), a highly sensitive magnetic readout system.^64,65^ Magnetic assays with detection limits of up to 1 fM were developed in recent years, yet only through enzymatic reactions.^66–68^ Recently, we have successfully detected the viral genome of SARS-CoV-2 by disassembling clusters of MNPs using a nonenzymatic TMSD-based DNA circuit and reading out the corresponding change in the MPS signal.^25^ With a limit of detection (LoD) of 27 pM after 24 h of assay time, the current declustering-based assays are still unable to function in clinical POC settings, where a low detection limit and short assay time must be merged, thus keeping the magnetic assays from unlocking their unique potentials.

Here, we propose innovative nonenzymatic declustering-based assays for the detection of nucleic acids in solution by integrating mismatch-assisted toehold exchange (MATE) in magnetic diagnostics cascades. Toehold exchange and base-pair mismatches have individually been employed to improve the kinetics of TMSD-based circuits,^40–42^ yet their combination and usability in magnetic diagnostics circuits are completely unexplored. We demonstrate that MATE is a highly efficient means of recycling nucleic acid targets, amplifying the magnetic signal output, and accelerating the assay’s kinetics. Kinetics of target-dependent declustering of magnetic clusters can be greatly tuned by adding fuel strands that recycle the target via toehold exchange. Our experimental kinetic studies show the beneficial impact of a mismatched base-pair on the declustering rate and equilibrium state. OxDNA coarse-grained model^69,70^ simulations on the effects of mismatch position on dissociation/declustering kinetics support the experimental observations. The mismatch-mediated duplex destabilization enhances the opening rate of the allosteric toehold, hybridization of fuel strands, and recycling of the target significantly. Our novel MATE cascades have a LoD of 22.2 pM after a total assay time of 2 h, as determined from MPS measurements. Our work demonstrates that MATE accelerates the kinetics of nonenzymatic magnetic DNA assays drastically while improving the LoD, taking a major leap toward translating magnetic nanoparticle-based assays to the clinical POC settings.

## Results and Discussion

We integrated our MATE cascade into so-called declustering-based magnetic bioassays, which register magnetic signal gain upon dissociation of MNPs from preformed magnetic clusters as well as their disintegration into smaller clusters in response to TMSD by a DNA/RNA target.^35,61^ Through these declustering events, the overall cluster hydrodynamic size decreases, and Brownian magnetic relaxation processes become faster on the ensemble level, resulting in the magnetic signal gain at the specifically chosen excitation field frequency (see the Supporting Information (SI) for more details). We have observed over time that nonamplifying circuits lead to only partial declustering at low target concentrations (Figure 1a, left panel), providing a low overall signal gain, since the target strands cannot react further after one TMSD event is complete. TMSD (Figure 1b) relies on the accessibility of a toehold domain on a substrate strand (green, S), a single-stranded nucleic acid domain, where an invading strand (T) can attach to and initiate strand displacement. During strand displacement, the invader can form additional base-pairs with the substrate strand upon spontaneous opening or breathing of an adjacent base-pair between the incumbent (L) and the substrate. Doing so, the invader T displaces the incumbent until full hybridization between T and S and release of L.^21^ In TMSD reactions, which use toehold exchange, the incumbent strand L is not fully displaced by the invader T, and the last few base-pairs (orange) need to dissociate spontaneously. Upon successful dissociation, a secondary, allosteric toehold (orange) is formed, available for further binding and displacement reactions. The spontaneous dissociation may occur only if the number of remaining base-pairs between incumbent L and substrate S becomes sufficiently low as the duplex grows unstable.^37,71^

**Figure 1:**
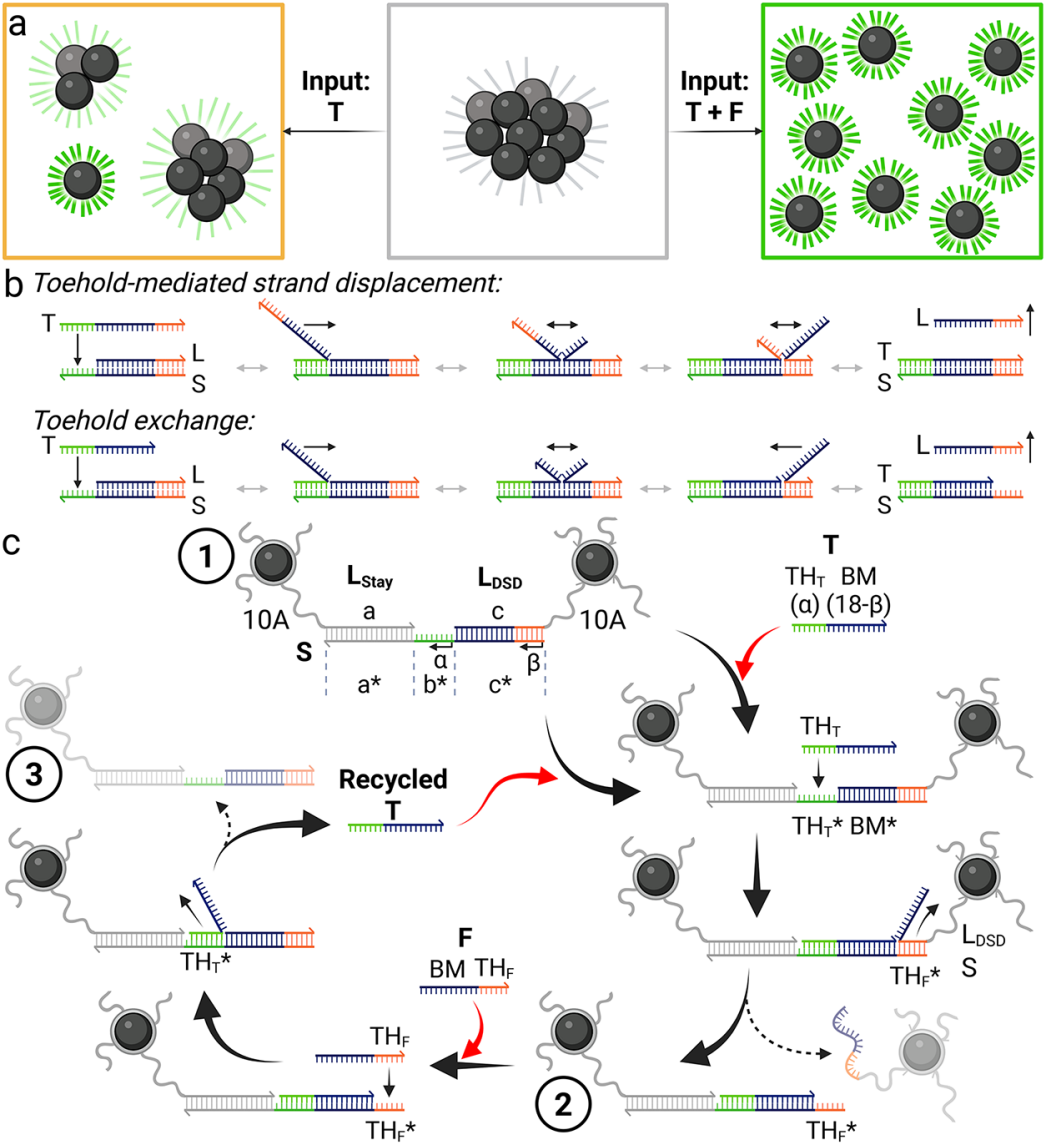
Scheme of MATE diagnostics cascades. (a) Scheme of declustering of magnetic clusters upon addition of target DNA (T) at low concentrations in the absence (middle → left panel) or in the presence (middle → right panel) of fuel DNA (F), leading to partial or full declustering, respectively. (b) Principle of TMSD with full displacement and toehold exchange. (c) MATE declustering cascade. First, T displaces L_DSD_. The last β bps of L_DSD_ dissociate spontaneously, resulting in a release of an MNP from magnetic clusters (stage 1 → 2). Afterward, TH_F_* becomes accessible for backward TMSD reactions, wherein F docks, branch migrates and releases T (stage 2 → 3). Next, the recycled T initiates the next cycle, leading to further declustering reactions, thus amplifying the total signal gain at low T concentrations. The magnetic clusters are irregularly shaped 3D structures, here shown as dimers for simplicity. Created in BioRender.

A typical MATE assay comprises preformed magnetic clusters and target (T) and fuel (F) strands. The clusters are formed by combining two types of BNF80-MNPs (see SI for synthesis procedures), one type labeled with label DNA Stay (L_Stay_, domain a) and the other labeled with label DNA DSD (DNA strand displacement, L_DSD_, domain c), with substrate DNA strands (S) (Figure 1c, stage 1, and Tables S1 and S2 for the sequences). S consists of domains a* and c*, complementary to the label DNA, as well as toehold domain b*, where the toehold TH_T_ of T can attach to. Strand T consists of TH_T_, α nucleotides, and a branch migration domain (BM). BM is equivalent to domain c, reduced by β base-pairs (bps) from the MNP-proximal 3′ end. In a MATE declustering cycle, T displaces L_DSD_ up to the last β bps upon binding to TH_T_* and branch migration. An MNP is then detached from the clusters if the β bps dissociate spontaneously (stage 2). The released MNP and the magnetic ensemble, reduced in hydrodynamic size, increase the magnetic signal output, which can be read out as a gain in magnetic susceptibility values (see Figure S1). Simultaneously, the previously hidden domain TH_F_* is now accessible for F. Strand F attaches to TH_F_* and displaces domain BM between T and S in a reverse displacement reaction. Upon completion, α base-pairs of TH_T_ dissociate spontaneously from S, completing one MATE cycle by releasing T and feeding it into the next cycle (stage 3). In the following paragraphs, we discuss how the domain TH_T_* and TH_F_* lengths, minor base-pair variations, as well as DNA and salt concentrations influence the assay’s kinetics.

### Implementing a Mismatch in TH_F_ Enhances Signal Gain Most If Placed at the First Base-Pair of Spontaneous Dissociation

We first studied how the declustering rate depends on the number of base-pairs in TH_F_ (β), which need to detach spontaneously, by varying β from 5 to 8 bp, while keeping α = 7 bp. A systematic reduction in the rate was observed by increasing the length of β compared to full displacement of the L_DSD_ (β = 0 bp, see Figure S2), aligning with the literature.^37,72^ While the declustering with β = 6 bp causes a visible magnetic signal change, the overall declustering rate is reduced as a result of slow spontaneous dissociation, thus limiting the signal gain. Consequently, we considered how implementing a mismatched base-pair between S and L_DSD_ would increase the declustering rate. We inserted one noncanonical base-pair at different positions in the spontaneous dissociation domain on S TH_F_* while keeping the rest the same, which locally destabilizes the duplex between L_DSD_ and S*x* (mismatches are highlighted in red in Figure 2a). The mismatch identities were chosen based on NUPACK simulations to ensure full hybridization of the MNP-proximal end of the duplex, as different mismatched base-pairs destabilize DNA duplexes differently^43,73,74^ (see Table S2 for sequences and estimated energies of the respective duplexes).

**Figure 2:**
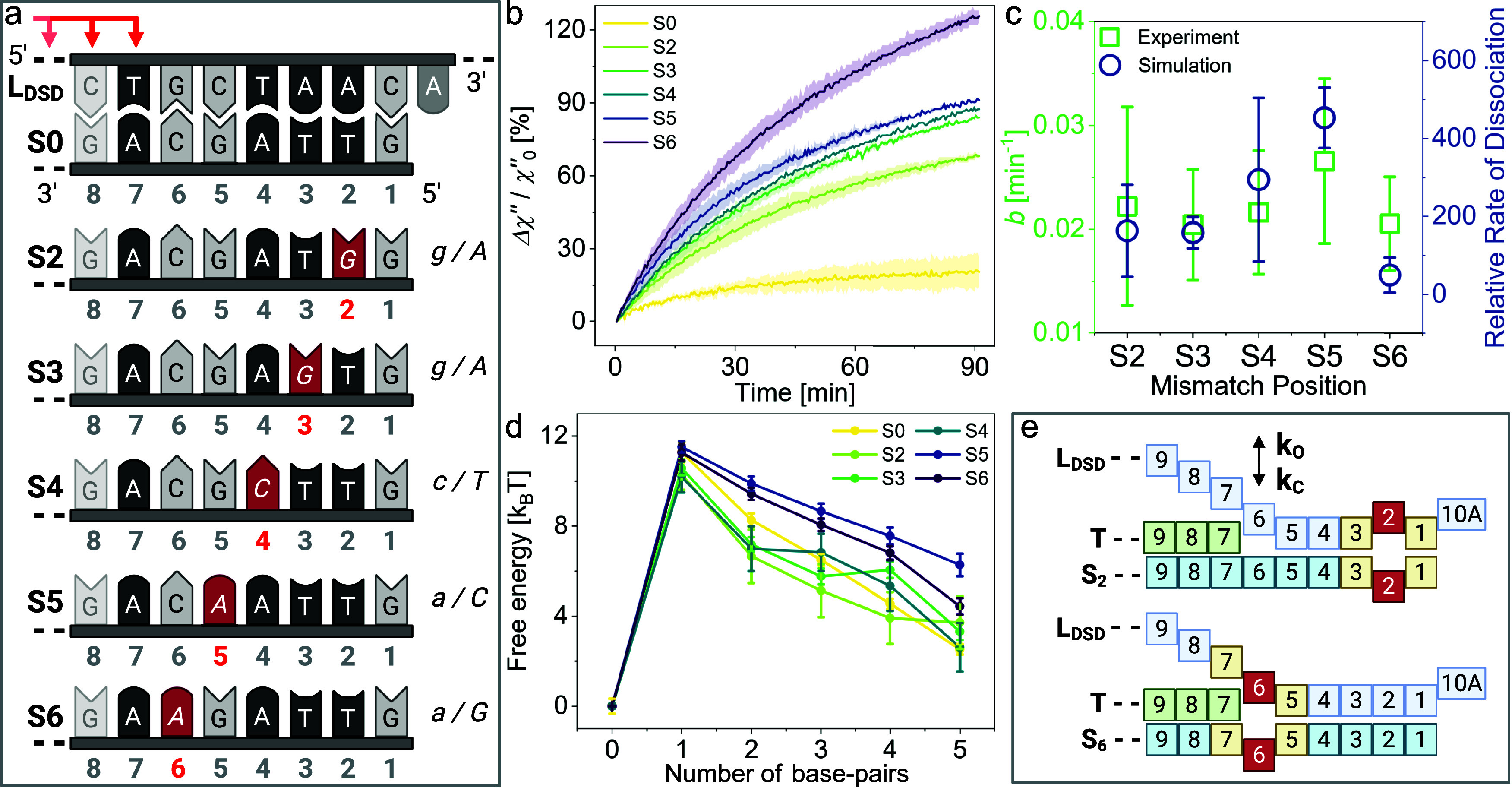
Declustering rate and signal gain as a function of spontaneous dissociation in response to T: (a) Base-pairs at the MNP-proximal end of the L_DSD_:S duplex for full complementarity between S and L_DSD_ (S0) and with mismatches at different positions *x* (S*x*), counted from the MNP-proximal end. (b) Experimental data measured with alternating current susceptometry (ACS) at 120 Hz and 0.5 mT/μ_0_. Relative change in the imaginary part of complex ac magnetic susceptibility Δχ″ over time for assays on clusters with different mismatches (100 mM Mg^2+^, 4 nM S*x*, 1 nM T (α = 7 bp, β = 6 bp)), calculated from two independent replica, displayed 1σ error shade. (c) Relative dissociation rate (with respect to no mismatch case S0) as measured in oxDNA forward flux sampling for different mismatch positions, compared to the saturation rate *b* fitted to experiments. (d) Free-energy profile for strand dissociation for different mismatch positions, as obtained from oxDNA simulations. (e) Visualization of destabilizing effect of mismatch (red) at position 2 (S2) and 6 (S6), complementary base-pairs are displayed in yellow (destabilized by mismatch), green (T), and blue (S, L_DSD_). Panels (a) and (e) were created in BioRender.

To understand how the mismatch position influences the declustering rate and eventual availability of the toehold TH_F_*, we first performed magnetic assays in the absence of F. The kinetics of declustering reactions were monitored by measuring the imaginary part χ″ values of complex AC magnetic susceptibility^75,76^ at the excitation frequency of 120 Hz and field strength of 0.5 mT/μ_0_, chosen based on magnetic measurements at different conditions (see Figure S1). Once declustering occurs, MNPs are released, and the overall hydrodynamic size of the magnetic clusters decreases, both increasing χ″. Looking at the relative change in χ″ (eq S4) measured over 90 min at 1 nM T, we observed that the gain in magnetic signal strongly depends on the position of the mismatch on S (Figure 2b), implying different declustering rates (Figure 2c). In the case of a mismatch-free duplex (S0), the reaction saturates quickly with only a small signal gain, indicating inhibited declustering at S0. The closer the mismatch is to the last base-pair between T and S (bp 7), the more declustering reactions that occur.

To quantify the declustering processes, we fitted the experimental data to an exponential function given by

1 $$y_{1}\left(t\right)=A\cdot \left(1-\exp\left(-b\cdot t\right)\right)$$

with time *t* in min, saturation rate *b* in min^–1^, and total signal gain *A* in % (see Table S3 for all values). Parameter *A* increases by placing the mismatch closer to where T ends, with cases S6 and S0 showing the highest and lowest values, respectively. The average saturation rate, *b*, indicating the rate at which the assay completes, shows an intriguing and rather unexpected trend (Figure 2c). It increases from S2 to S5 and then drops for S6. S5 shows the highest *b* value of 0.027 min^–1^. While the circuits with mismatch S6 reach their thermodynamic equilibrium after a longer time with *b* = 0.021 min^–1^, its *A* = 147.9% value is 7.1-fold higher than the case of full complementarity S0 (*A* = 20.9%, see Table S3 and Figure S3 for fit parameters prior to averaging).

To further investigate the mechanism of spontaneous dissociation of L_DSD_ (the step immediately preceding stage 2 shown in Figure 1b), we used oxDNA simulations. We obtained free-energy profiles as a function of the number of base-pairs formed for different positions of the mismatch between the substrate and the dissociating strands. The profiles (Figure 2d) show that the mismatch position 5 (S5) is the most destabilizing, as seen by the relative free-energy difference between the fully unbound (0 bp) and fully bound strand (5 bp). Other mismatch positions were estimated from the profile to be about 1 to 4 *k*_B_ *T* more stable in the bound state than the S5 one. This was also confirmed with our forward flux sampling (see Figure 2c), where the dissociation with a mismatch in position 5 was the fastest, aligning well with our experimentally derived saturation rates *b*. Thus, our dissociation study illustrates how mismatch position can be used to fine-tune spontaneous dissociation kinetics in strand displacement cascades.

Relevant for our biosensing application is the total signal gain *A* at a certain run time. If the system reaches its thermodynamic equilibrium quickly (high *b* value), but does not yield a significant change in signal, it limits the potential LoD and is therefore not suitable for the detection of low numbers of target DNA. Our experimental ACS results clearly show that the total signal gain *A* and the correlating overall declustering with spontaneous dissociation of 6 bps are increased significantly if a mismatch is positioned adjacent to the last base-pair of the hybridized T. Two mechanisms may explain why positioning a mismatch further away from the BM domain reduces the overall declustering. One explanation might be that the TH_F_ domain of L_DSD_ detaches from S*x* and quickly reattaches to a free TH_F_* domain on S*x*. As a result, MNPs are always seen in the clustered state, and thus, no magnetic signal Δχ″ is gained. To test this hypothesis, we added T with blocker strands to the cascade, which would attach to the TH_F_* and TH_F_ domains once those are accessible after spontaneous dissociation of L_DSD_. We observed no significant change in the declustering behavior (see Figure S4 for data and Table S4 for sequences). Therefore, we argue that a more plausible reason for limited declustering behavior is the difference in the initial detachment of TH_F_ once T has finished the branch migration step. The toehold exchange reaction is a stochastic process based on forward and backward steps of each base-pair involved. Once T is fully hybridized to S, the detachment of TH_F_ depends on the opening *k*_O_ and closing *k*_C_ rates of the last 6 bps of L_DSD_. If base-pair 6 opens, the remaining 5 bps are less stable and are increasingly prone to spontaneous dissociation. Conversely, if base-pair 6 is connected to S, L_DSD_ can initiate a reverse TMSD reaction and displace T. This will, in turn, limit the declustering processes and the magnetic signal gain Δχ″, as schematically shown in Figure 2e with the mismatch colored red and the adjacent destabilized nearest neighbors (NNs) in yellow. Both the opening rate *k*_O_ and closing rate *k*_C_ depend on the local energy landscape of the duplex. The presence of a mismatch destabilizes its immediate location and its NNs and deforms the helical structure for up to a few neighboring base-pairs.^73^ In the proximity of a mismatch, the opening rate *k*_O_ outweighs the closing rate *k*_C_, since the formation and enclosing of a mismatch is energetically unfavorable.^40,44,77,78^ While a mismatch at position 2 (S2) enhances *k*_O_ of TH_F_ once the detaching branch has reached the last base-pairs (yellow and red), it has a marginal influence on *k*_O_ at position 6 (blue). Therefore, the mismatch in S2 inhibits the backward pathway only minimally due to the complementary domain between S2 and L_DSD_ being reduced to 4 consecutive base-pairs. The closer the mismatch is shifted to position 6, the earlier the undesired backward path by L_DSD_ is hindered. At position 6 (S6), an energetic barrier has to be overcome to form a base-pair at the position of the mismatch (red)^77,79^ and be closed long enough for the base-pair between T and S at position 7 (green) to breathe and allow initiation of a reverse TMSD reaction (yellow). The probability for this event to take place is significantly lower than that for TH_F_ to detach once T has completed its branch migration. Additionally, the forward branch migration of the last base-pairs of T is increased due to the local destabilization caused by the mismatch at position 6. One further influential factor could be the distance of the mismatch to the duplex end. A mismatch may have difficulties to form a closed base-pair if it is enclosed by numerous base-pairs that are forcing a helix-structure, while a lower number of base-pairs adjacent to a mismatch closer to the duplex end can deviate more easily from the helix-structure to adapt to the mismatch-induced deformation.

### Recycling Efficiency of Magnetic Cascades Increases with Mg^2+^ and the Length of Allosteric TH_F_

Moving forward with our investigations, we realized that adding 100 mM Mg^2+^ to the cascade improves the declustering up to 17-fold by enhancing hybridization of TH_T_, similar to what has been reported by Yang et al.^80^ Importantly, Mg^2+^does not impede the spontaneous dissociation of TH_F_ (Figure S5).

In toehold exchange-based cascades, the difference in base-pairs of TH_T_ and TH_F_ (α – β) plays an equally important role as the length of each toehold domain,^37^ yet it is completely unknown how this reflects in magnetic signal amplification cascades like ours. To shed light on this aspect, we varied the length α of TH_T_ from 5 to 7 bp, while keeping β = 6 bp, and measured Δχ″ in the presence of T. The overall signal change increases with (α – β) for both 0 and 100 mM Mg^2+^ (Figure S6). Having more base-pairs in TH_T_ (α = 7 bp, α – β = +1 bp) enhances the toehold formation and favors the hybridization of T over L_DSD_ to S thermodynamically due to the gain of one base-pair in the duplex, both effects improving the subsequent declustering rate. In this study, we opted to continue with a TH_T_ length of α = 7 bp to enable the fast and reliable opening of TH_F_*.

We then sought to understand how efficiently our MATE cascade could recycle T and feed it back into the next declustering cycle if input F was added. F binds to TH_F_* if it is available upon the completion of declustering (stage 2 in Figure 1c). Hence, adding F to the MATE cascade recycles T in a reverse TMSD pathway (stage 2 → stage 3), feeds T back into the next cycle, and completes one signal amplification cycle. We tested two different lengths β of TH_F_ in the presence and absence of a mismatch in TH_F_ (Figure S7). Over three independent sets of experiments with α = 7 bp, β = 6 bp showed significantly higher recycling capabilities compared to β = 5 bp. The reverse reaction is offered a longer, more stable toehold for β = 6 bp, enabling F to initiate the recycling of T more efficiently while losing just one instead of two base-pairs during the reverse TMSD where T is displaced by F. These two factors align to enhance the reverse TMSD reaction and the consequent recycling of T. These effects are even stronger in the presence of a mismatch in TH_F_, highlighting the importance of a longer TH_F_ domain for enhanced recycling. Fundamental event during TMSD is the formation of the toehold, which becomes more stable for each additional base-pair that is involved, up to a maximum of 6 or 7 bp.^21^ A stable toehold increases the rate of starting the branch migration process and the chance for the successful completion of a TMSD reaction. Besides the stability of the toehold, the number of base-pairs in a duplex is the other driving factor in the TMSD reactions since more base-pairs in a DNA complex are favorable thermodynamically. In the toehold exchange, three successive events need to take place: the toehold formation at the first toehold, the branch migration, and the spontaneous dissociation of the second toehold. The first event is enhanced if the first toehold is long for stable hybridization of the invader and branch migration initiation. The spontaneous dissociation is enhanced if the second toehold domain contains few base-pairs, so the incumbent may detach quickly. In the case of our magnetic cascade, we require a forward toehold exchange (Figure 1c, stage 1 → stage 2) to open TH_F_* with invader T and a controlled reverse toehold exchange (stage 2 → stage 3) to recycle the invading T with the fuel F. Therefore, we require all three events in both directions to be sufficiently efficient. Our investigations in our novel magnetic cascade match the reported optimal value of α – β = 7 bp – 6 bp = +1 bp.^37^

### MATE Increases Target Recycling by 14-Fold

Previously, we studied the influence of the mismatch position on the opening of TH_F_ (Figure 2b) by adding only T, modulating the magnetic system from stage 1 to stage 2 (Figure 1c). The findings motivated us to look into how adding F to the cascade enables the reaction pathway from stage 1 to stage 2 and stage 3 (Figure 3a). We investigated the signal gain for all mismatch positions at α = 7 bp and β = 6 bp. The trend of Δχ″ over 90 min between the different mismatch locations increases nonchronologically via 0 ≪ 4 ≤ 2 < 5 ≪ 3 < 6 (Figure 3b). The magnetic signal for an assay with complete MATE amplification is enhanced by 5.2-fold from Δχ″ = 34% for S0 to Δχ″ = 176% for S2. Although F is present in excess (100 nM F, 4 nM S, and 1 nM T), the disassembly of S0-based, mismatch-free clusters is significantly restrained due to the slow and limited opening of the allosteric TH_F_*. The Δχ″ increases most for S6 by 14.4-fold to 490%. The cascade of S0-based clusters reaches saturation after 1.5–2 h, while the Δχ″ of the samples with mismatched substrates still increases significantly after 1.5 h, indicating that the declustering and signal amplification are still in progress. This behavior is based on the cooperation of the spontaneous dissociation of TH_F_ after TMSD of T and leakage, which is declustering caused by F invading at breathing base-pairs at the mismatch location, changing the clusters from stage 1 directly to stage 3 (Figure 1c).

**Figure 3:**
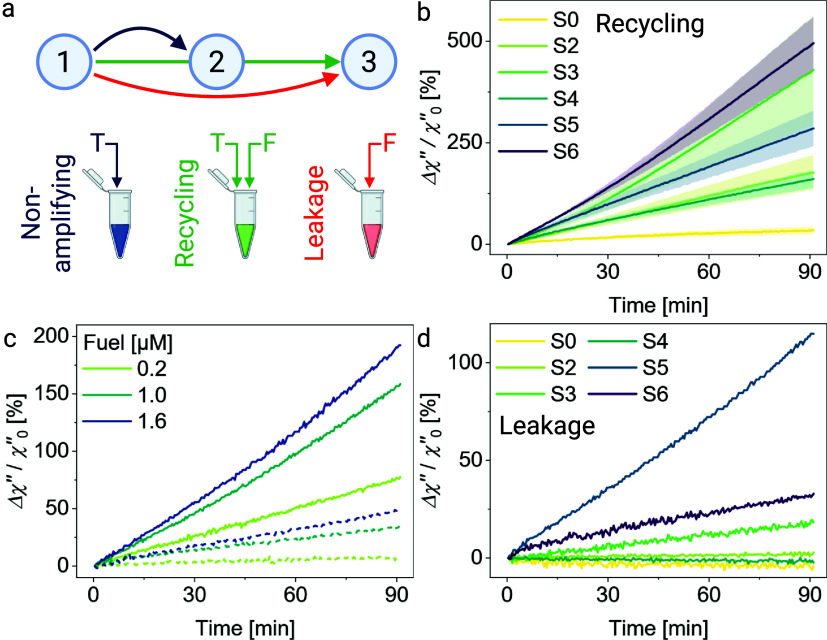
Magnetic signal amplification via the recycling of T by F. (a) Schematic presentation of different declustering pathways depending on the DNA strands added to the clusters. Stages named here correspond to the stages shown in Figure 1. (b) Δχ″ for all mismatch positions S*x* (1 nM T (α = 7 bp, β = 6 bp), 100 nM Fx, complementary to S*x* (4 nM)) measured by ACS. The curves are the average of three independent sets of samples (1σ error shades). (c) Δχ″ on S6 for increasing Fuel concentrations with 0.25 nM (solid) and without T (dashed). (d) Δχ″ for different mismatch positions of S*x* at 1 μM Fx in the absence of T (leakage). All measurements contained 100 mM Mg^2+^. Panel (a) was created in BioRender.

We next explored to what extent the recycling of the target and its reuse in subsequent declustering reactions can be accelerated by concentration-driven TMSD.^21^ To do so, we varied the F concentration from 4 nM (F/S 1:1) to 100 nM (F/S 25:1) and looked at how the magnetic signal changes accordingly (Figure S8). Due to the loss of a base-pair in the reverse TMSD path during recycling (β – α = 6–7 = −1 bp), the hybridization product SF is slightly less favorable than ST, requiring oversaturation of F over S. We measured Δχ″ for clusters with a mismatch at position 6 (S6) at three F concentrations (Figure 3c) in the presence (solid lines) and absence (dashed lines) of 0.25 nM target. After 90 min, Δχ″ increases from 77 to 192% and 9 to 49% by increasing F concentration from 0.2 to 1.6 μM in the presence and absence of T, respectively. Admittedly, we observed some declustering in the absence of T, an effect known as leakage (dashed lines in Figure 3c), which increases with the F concentration. The leakage is presumably due to enhanced breathing of the domain TH_F_ resulting from duplex destabilization by the mismatch.

### Highest Opening Rate of TH_F_* by T Does Not Entail Highest Leakage

Moving toward establishing an assay workflow using MATE, it is important to understand the origin of the leakage. To shed light on this critical topic, we first looked at how the leakage depends on the mismatch position at high F concentrations, as different mismatches destabilize the duplex between L_DSD_ and S*x* differently. We recorded Δχ″ for all mismatches by adding 1 μM Fx to different MATE cascades (Figure 3d) in the absence of T. We observed −5.3, −2.3, and +1.8% change in Δχ″ for S0, S4, and S2, respectively, over 90 min incubation time, indicating no significant leakage for these designs. The situation for the other designs is quite different. The S3 and S6 show 19.1 and 31.2% signal change leakage. Most significant is the leakage for S5 with 114.8% increase in Δχ″. We observed a similar trend in leakage for the mismatches studied for other concentrations of F, with the effect increasing with concentration (Figure S9).

To understand these results better, we calculated the free-energy Δ*G* values (Table 1) of the DNA complexes in clusters (stage 1 in Figure 1c) via NUPACK. We found that the difference in Δ*G* increases by placing the mismatch further away from the MNP-proximal end and climaxes for S5, matching the experimental observations (Figure 3d). Based on the differences in Δ*G*, we expected S3 to show leakage behavior similar to that of S2 and S4, which is not the case. Inspecting the NN of the mismatched base-pair, we notice that the mismatch at S3 is surrounded by two A–T pairs, which are more likely to breathe than G–C pairs.^73^ In S2 and S4, the mismatch is enclosed between one A–T pair and one G–C pair. While the mismatches at positions 5 and 6 are adjacent to one G–C NN, the mismatched base-pair replaces a G–C pair instead of an A–T pair (as for S2 and S4) and reduces the GC-content in the TH_F_ domain. Therefore, we hypothesize that not only are the base-pairs directly adjacent to the mismatch relevant for the duplex stability but also bases further away from the mismatch play an important role. The leakage for S5 may additionally be higher due to the A–C mismatch identity chosen in this study. Oliveira et al. observed A–C pairs lacking strong hydrogen bonds,^43^ and Rossetti et al. reported A–C to show the highest breathing portions of the mismatches studied in our work.^73^ Interestingly, NUPACK calculations show a 60% probability for the last base-pair of S2 to be closed (see Figure S10). This would entail that the last two base-pairs would be open for nearly half of the time, which would lead to significant leakage. Oliveira et al. reported that the mismatch A–G within the sequence AaC/TgG exhibits a double hydrogen bond and is therefore exceptionally stable,^43^ which may explain the observed low leakage for S2. We acknowledge the influence of the identity of the mismatch as well as the NN on the stability of the duplex.^43,74^ The sheer number of mismatch-NN combinations goes beyond the scope and capability of this research.

**Table 1:** Sequences of the Mismatch and Its NN^a^

substrate S*x*	mismatch + NN identities *x* ± 1	Δ*G* [kcal/mol]	Δ*G* (S0) – Δ*G* (S*x*) [kcal/mol]
S0	-	–59.77	±0
S2	AAC/TgG	–57.14	+2.63
S3	TAA/AgT	–56.39	+3.38
S4	CTA/GcT	–56.08	+3.69
S5	GCT/CaA	–54.32	+5.45
S6	TGC/AaG	–54.93	+4.84

a Mismatched base at position x as a lower-case letter, Δ*G* simulated with NUPACK for 150 mM Na^+^, 100 mM Mg^2+^, and 5 nM Oligos (L_DSD_, L_Stay_, S*x*) at 25 °C.

### Local Duplex Stabilization around Mismatch Regulates Leakage and Spontaneous Dissociation of TH_F_

The peculiar correlation between the mismatch position and leakage motivated us to explore how the leakage is modulated by duplex stabilization around the mismatch, with a focus on S6, as it shows the highest spontaneous dissociation of the TH_F_ domain and comparatively low leakage. To do so, we modified S6 in the TH_F_ domain in two specific ways. First, we replaced a single nucleotide with a locked nucleic acid (LNA) at different neighboring positions without changing the base identity (Table 2 and Figure 4a) and looked at how the stabilizing nature of the LNA^81,82^ influences the leakage and target-catalyzed declustering rates. The ribose ring of LNAs exhibits an additional methylene bridge bond, which increases the base-stacking interactions, hardens the strand backbone, and increases the duplex thermal stability, reducing the opening rates of LNA–DNA base-pairs.^82^ Further, we modified the last two base-pairs from the MNP-proximal end by inserting a CG clamp (see Figure 4a and Table S5). We compared the leakage by monitoring Δχ″ over 90 min by adding only F to the MATE cascade (Figure 4b). Remarkably, the cascade shows a higher change in Δχ″ and therefore faster declustering for the CG variation than for the basic version, suggesting that the leakage does not stem from the duplex end and that the increased CG content in TH_F_ (33.3 → 50%) facilitates hybridization of F to the TH_F_* domain. Placing an LNA at positions 2 and 5 stabilizes the L_DSD_:S duplex structure only slightly and reduces the leakage marginally. The behavior is very different when the LNA is at position 7, where a significant drop in leakage was observed (Table 2). Our results strongly suggest that the leakage stems mainly from the A–T pair at position 7. We hypothesize that the mismatch destabilizes its NN asymmetrically, with the destabilization and breathing of the A–G mismatch at position 6 being more prominent toward the A–T base-pair than toward the G–C NN. Therefore, employing an LNA at position 7 inhibits the leakage by F most effectively.

**Figure 4:**
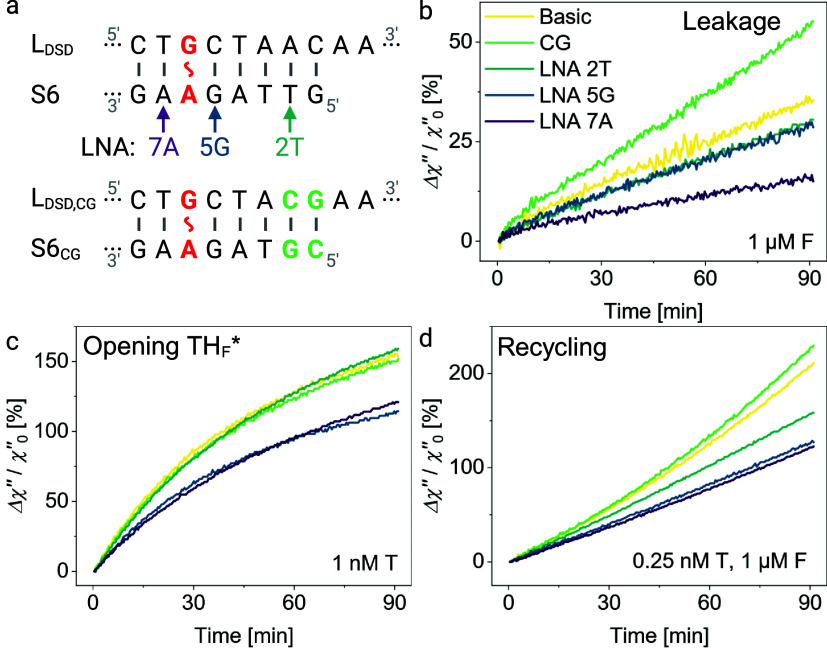
Reducing leakage with locked nucleic acids. (a) Sequence changes were made to vary the local duplex stability. Changes in magnetic signal over 90 min for (b) leakage-based declustering (1 μM F) in the absence of T, (c) declustering at 1 nM T in the absence of F, and (d) the whole MATE cascade at 0.25 nM T and 1 μM F. Panel (a) was created in BioRender.

**Table 2:** Magnetic Signal Gain Δχ″ over 90 Min for Different TH_F_ Modifications^a^

sequence variation	last 8 nt of S6 (3′ → 5′)	leakage [%] 1 μM F	no recycling [%] 1 nM T	recycling [%] 0.25 nM T, 1 μM F	leakage/recycling [%]
Basic	··· GAaGATTG	35.5	155.4	207.4	17.11
CG	··· GAaGAT**GC**	54.2	150.0	228.1	23.76
LNA 2T	··· GAaGAT{T}G	30.5	159.1	157.1	19.41
LNA 5G	··· GAa{G}ATTG	29.2	114.8	126.9	23.01
LNA 7A	··· G{A}aGATTG	15.3	120.9	121.6	12.58

a Varying substrate stability from the basic concept (S6) by addition of an LNA *yz* (*y*: position from MNP-proximal duplex end, *z*: base identity) marked with {···}, as well as a CG clamp, mismatch as a lowercase letter. Δχ″ after 90 min of declustering with F (leakage), with T (no recycling), and with fully functional MATE recycling and amplification. Ratio of Δχ″ in leakage relative to that in recycling.

Next, we investigated the influence of the duplex modifications on the spontaneous dissociation since this effect is at the core of MATE cascades. We observed no significant change in Δχ″ for the CG variation and the LNA at position 2 (Figure 4c). The declustering decreases for the LNAs in positions 5 and 7. Therefore, we argue that the last two base-pairs do not impact the spontaneous dissociation of TH_F_ in contrast to the NNs of the mismatch. The LNAs at positions 5 and 7 reduce the opening rate of TH_F_* noticeably as the incumbent is stabilized around the mismatch.

Highly relevant for our magnetic DNA cascade is the full MATE cascade amplification, where T is recycled back into the circuit by F (Figure 4d). A minimal increase in Δχ″ can be observed for the CG variant (Table 2; δ­(Δχ″/χ″_0_) = +9.9%, calculated using eq S6), which is plausibly due to increased leakage (+52.7%) rather than a better functioning cascade. The magnetic signal gain Δχ″ and its underlying declustering efficiency are reduced for all tested LNAs due to the reduction in leakage and spontaneous dissociation. The LNA in position 7 is the only variation that features a reduction in leakage to recycling ratio from 17.11% (Basic) to 12.58% (LNA 7A) (Table 2) and is therefore the most favorable S6 variant for diagnostic applications using the MATE cascade.

### MATE-Based Declustering Increases Linearly with T and Exponentially with F

By combining all of the optimal conditions established so far, here, we work toward realizing MATE cascades for diagnostics. Up to this stage, we primarily looked at how a MATE cascade responds to single base-pair variations. Here, we asked how a full MATE cascade functions if the F and T concentrations are varied over a broad range. First, we varied the F concentration from 0 to 4 μM at 0.25 nM T and monitored changes in χ″ (Figure 5a). Examining the relative change in χ″ at 150 min, we monitored saturation in signal gain for high F concentrations (Figure S11) and refrained from increasing it to more than 4 μM, as the difference in signal gain between the recycled reaction (with T and F) and the leakage (with only F) stagnates with F concentration. The comparably low target concentration was chosen to simulate the performance of our assay and show the relevance of MATE-based amplification for clinical samples with a low target DNA concentration. For all samples, we observe a linear increase in χ″, except for the case of 0 μM F, which saturates within the recorded time due to the absence of target recycling and signal amplification. By increasing the fuel concentration, a much greater change in signal χ″ after a shorter time was observed, with the steepest rise for 4 μM F. To estimate the reaction rate, we fitted the measured data to a simple linear function given by

2 $$y_{2}\left(t\right)=d\cdot t+e$$

with *d* being the slope in min^–1^ and *e* being the offset in a.u., compensating for minor MNP concentration differences. The curve at 0 μM F was fitted only up to the first 30 min, where χ″ increased linearly.

**Figure 5:**
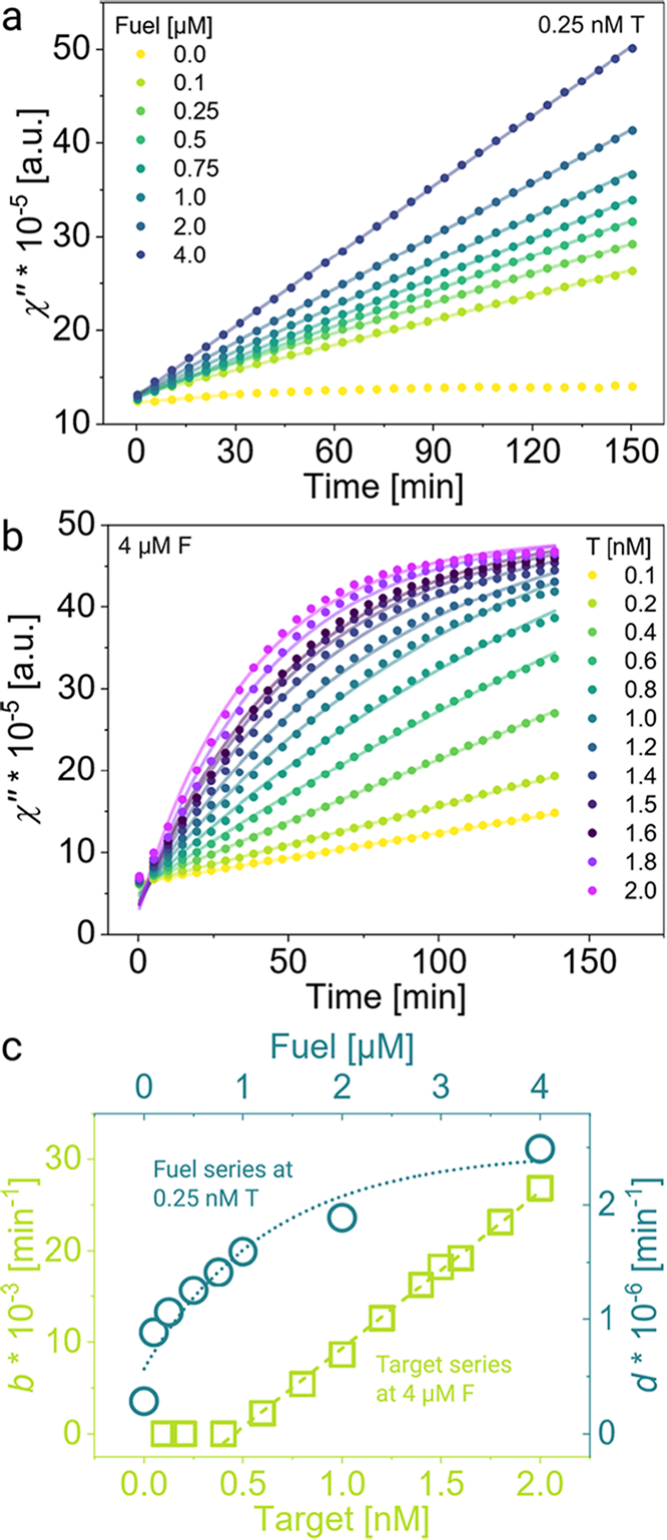
Target and fuel concentration-dependent signal amplification. (a) χ″ measured at different F concentrations at 0.25 nM T, solid lines were fit with eq 2. (b) χ″ at different T concentrations at 4 μM F, solid lines were fit with eq 3. Measurement data were fitted first and then interpolated for better display (circles). (c) Parameters *b* and *d* from fit functions over respective concentration; dashed lines were plotted via eq 2 (green, target series) and 3 (turquoise, fuel series). All experiments were executed with α = 7 bp, β = 6 bp, S6, LNA variation in position 7, and 100 mM Mg^2+^. All clusters within one panel are from one preparation batch, enabling the plotting of magnetic susceptibility χ″ as recorded.

In another set of experiments, we recorded changes in χ″ by varying the T concentration from 0.1 to 2 nM at a constant [F] of 4 μM (Figure 5b). The kinetics of declustering and signal amplification seem very different compared to that when the *F* concentration was increased. The χ″ increases faster with [T]. Higher T concentrations lead to higher opening rates of TH_F_* and efficient hybridization of F strands to fully accessible allosteric toehold TH_F_*. This, in turn, leads to more target recycling and a complete declustering of magnetic clusters after shorter times. At [T] > 1 nM, χ″ rises exponentially and saturates within the measured time, yet the saturation onset shifts to earlier times by increasing the T concentration further. Interestingly, we can observe a slight phase lag for the investigated concentrations (see Figure S12 for an enlarged view of the unprocessed measurement data), which could indicate cooperativity between F and T on disassembling the magnetic clusters. Furthermore, we observed a minimal increase in starting value χ″_0_ (circles) due to an inevitable short time delay between mixing the sample and starting the measurement, during which the declustering reaction is already initiated. The variation between the starting points is insignificant compared to the overall signal change after 150 min. We fitted these curves to a modified version of eq 1

3 $$y_{3}\left(t\right)=A\cdot \left(1-\exp\left(-b\cdot t\right)\right)+c$$

with parameter *c* being the offset of the non-normalized data χ″ (in a.u.), which shifts slightly to lower values for rising [T] due to reduced fit accuracy for the initial data points.

The declustering rates, *d* and *b*, obtained from these two concentration-dependent series reveal interesting features (Figure 5c). By increasing [F] up to 16.000-fold of [T], *d* increases exponentially as a function of [F]. At first, upon adjusting [F] from 0 to 0.1 μM, *d* soars due to the recycling of a low number of T strands. Next, it saturates at high [F], strongly suggesting that there is an upper limit for the impact of F on the declustering kinetics since the MATE cascade is catalyzed by T. In the experimental series, in which [T] was varied at 4 μM F, very different kinetics were observed (Figure 5b). A saturation in signal can be seen for [T] > 1 nM, which indicates that no more single MNPs can be generated in the cascade. The rates *b* obtained for this data set increase linearly with [T] at 4 μM F (first two data points excluded) since the MATE cascade is catalyzed by T.

To better understand these data, we broke down the MATE cascade into two reactions at the elementary steps

$$S+T\overset{k_{1}}{\to }ST,1st\;\mathrm{reaction}$$

$$\mathrm{ST}+F\overset{k_{2}}{\to }\mathrm{SF}+T,2\mathrm{nd}\;\mathrm{reaction}$$

with ST being the declustering product (i.e., MNPs), SF being the product after recycling of T, and *k*_1_ and *k*_2_ being second-order rate constants. The first reaction corresponds to declustering and magnetic signal gain, where the cascade transits from stage 1 to 2 (Figure 1b). The second reaction stands for the recycling of T by F as the cascade moves from stage 2 to stage 3. Considering these two reactions, we can see that the magnetic signal gain comes from the formation of ST in the first reaction. In other words, the consumption of ST in the second reaction does not directly change the signal since no MNP is released. Though consumption of ST does not increase the magnetic signal, the second reaction recycles T strands, which will be consumed in the first reaction pathways, influencing *k*_1_. As we do not have any means to separately determine *k*_1_ and *k*_2_, we discuss the reaction rates qualitatively. In the experiments in which we varied [F] at 0.25 nM T, we observed no signal saturation (Figure 5a), indicating a continuous generation of ST over the monitored time. In combination with the linear dependence of kinetic rate *b* on [T], this means that the first reaction is the bottleneck of the MATE cascades, wherein at low [T] limited amount of ST is produced and a low number of TH_F_* is catalyzed.

### MATE Diagnostic Cascades Have 12-Fold Shorter Assay Time than Existing Circuits

We then wondered how sensitive and rapid a diagnostic assay based on MATE would be. For these assays, we used the highly sensitive magnetic particle spectroscopy (MPS) technique and measured the magnetic samples after 2 h of incubation. Our custom, benchtop ImmunoMPS system magnetizes the sample at *f*_0_ = 590 Hz and 15 mT/μ_0_ and captures the sample’s magnetic response as higher odd harmonics of *f*_0_ within only 1 min of measurement time.^65^ The MPS harmonics spectrum is steeper for magnetic clusters and becomes shallower by their declustering to MNPs with smaller hydrodynamic sizes upon increasing T concentrations (Figure 6a). As the harmonics’ amplitudes scale linearly with the MNP concentration, here, we utilize the ratio of fifth to third harmonics (*HR*_53_, eq S5) as an MNP concentration-independent index to evaluate the declustering progress. An increase in the *HR*_53_ implies declustering.

**Figure 6:**
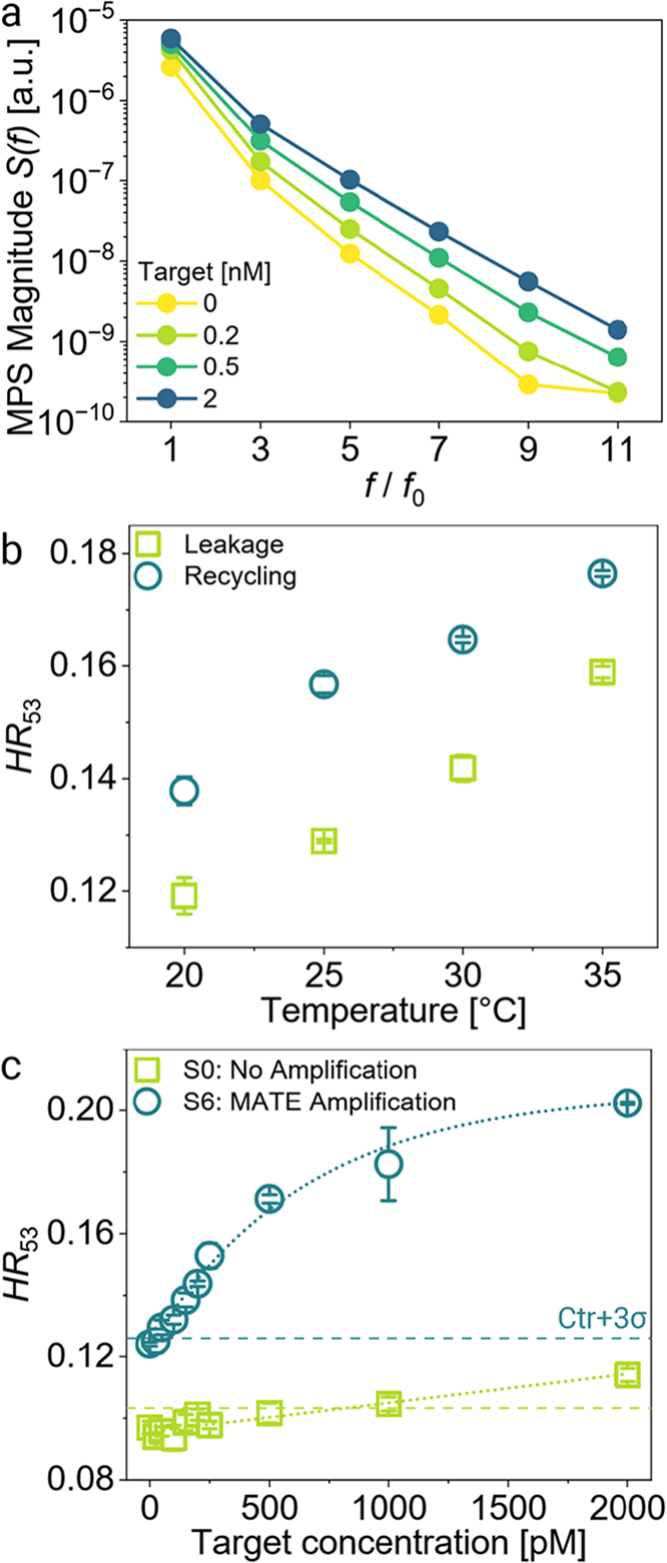
MPS measurements of MATE magnetic diagnostics cascades. Measurements were taken with our custom magnetic particle spectroscopy (MPS) setup, operating at 590 Hz and 15 mT/μ_0_, after 2 h of incubation at 25 °C under optimal conditions (100 mM Mg^2+^, α = 7 bp, β = 6 bp, S6, LNA variation in position 7, 4 μM F). (a) MPS harmonics spectrum at four different T concentrations taken from one series of S6 in panel c. (b) Harmonic ratio *HR*_53_ of MATE executed at increasing incubation temperatures for 0 nM (green squares) and 0.25 nM T (turquoise circles). (c) Dose–response curves for a nonamplifying magnetic assay with full TMSD (S0, α = 7 bp, β = 0 bp, 0 μM F, green squares) compared to an amplifying MATE cascade (turquoise circles). All *HR*_53_ results are the average of three independent sets of samples.

As temperature plays a significant role in TMSD reactions and our MATE is determined by spontaneous dissociation of the toehold domains and leakage, we varied incubation temperature for 0 pM and 0.25 nM T and measured the samples in triplicate (Figure 6b). The averaged *HR*_53_ increases with the incubation temperature for the target-catalyzed declustering (turquoise circles) but is even more so for the leakage (green squares). The greatest difference in *HR*_53_ can be observed at 25 °C. The relative change in *HR*_53_, meaning the signal increase relative to the leakage (calculated using eq S6), peaks at 25 °C at 21.5% and falls to 16.1% (30 °C), 15.5% (20 °C), and 11.1% (35 °C). While our MATE design works best at 25 °C, it can be utilized up to 35 °C over a range of temperatures.

We also varied the salt concentrations during incubation as clinical samples may vary in Na^+^ and Mg^2+^ concentrations. We observed no significant change by decreasing Na^+^ from 150 mM to 100 mM (Figure S13) at 100 mM Mg^2+^ but a great drop in performance by decreasing Mg^2+^ from 100 mM to 20 mM, while keeping Na^+^ at 150 mM. In light of developing an assay workflow for testing patient samples, the assay-relevant salt concentration can easily be adjusted by smart protocol design, as Mg^2+^ contents in bodily fluids are typically <1 mM,^83^ thus fluctuating the 100 mM Mg^2+^ concentrations required in our assays minimally.

Lastly, we determined the LoD of our MATE-based diagnostic cascade and compared it to our previous magnetic DNA assay.^25^ We combined clusters with 4 nM S6 (LNA at position 7) with 4 μM F, 100 mM Mg^2+^, and increasing target concentrations (0–2 nM, α = 7 bp, β = 6 bp). Additionally, we prepared a nonamplifying assay, containing 4 nM S0 and no fuel, as the target displaced all of L_DSD_ and no allosteric TH_F_* was offered (α = 7 bp, β = 0 bp). The samples were prepared in triplicate, incubated for 2 h at 25 °C, and then measured with our benchtop ImmunoMPS system. The *HR*_53_ of the MATE series (Figure 6c) starts at a low value (0.124) at 0 nM T and increases with T as a result of MNPs dissociating from the clusters. Once the clusters are fully disassembled into single MNPs, the magnetic signal saturates at *HR*_53_ = 0.202. The control samples without T (0 pM) define the cutoff line by applying the 3σ-criterion (dashed lines). The *HR*_53_ data were fitted with eq 3 for the amplifying MATE cascade (S6) and with eq 2 for the nonamplifying assay (S0). The intersection of the fit curve (dotted line) and the cutoff line determines the theoretical LoD of the nonamplifying case (S0) to be 820 pM (≈61.5 fmol), while our novel MATE cascade (S6) offers a sensitivity of 22.2 pM (≈1.665 fmol) after just 2 h, a 37 fold improved LoD. Compared to our previous work with an LoD of 27 pM after 24 h, the assay time was shortened by 12-fold.

## Conclusions

In this work, we proposed mismatch-assisted toehold exchange (MATE), a novel concept for a next-generation magnetic diagnostics cascade. The MATE cascade offers sensitive, simple, nonenzymatic, isothermal, amplification-free, and rapid diagnostics of nucleic acid sequences in solution. Here, we studied the kinetics of the cascade thoroughly by dissecting it into allosteric TH_F_ generation and target recycling. We showed that a longer allosteric TH_F_ leads to more efficient target recycling and higher signal gain. However, this initially reduces the rate and probability of spontaneous dissociation of the allosteric TH_F_. By implementing a mismatched base-pair in TH_F_, the reaction kinetics were enhanced significantly. The spontaneous dissociation rate was enhanced by a factor of ∼7 for a mismatch adjacent to the last base-pair of the invading target, leaving 5 consecutive base-pairs to detach spontaneously.

One major limitation of previous declustering-based magnetic assays is slow toehold hybridization. We solved this issue by adding divalent Mg^2+^ ions to the cascade, which accelerated the assay 17-fold, via stabilizing TH_T_ but not inhibiting the detachment of TH_F_. We improved the assay speed further via concentration-driven TMSD by adding more fuel strands and thereby gaining more signal at low target concentrations. Simultaneously, we observed mismatch-dependent leakage. Single LNA variations were inserted to restrict the leakage-based declustering, resulting in a favorable balance between unwanted leakage and the desired acceleration of the cascade. Our results show that the leakage in MATE cascades depends on the local design of the duplex in terms of the mismatch identity as well as its surrounding sequence. By performing MATE assays with our benchtop MPS and moving toward establishing simple and rapid assays, we determined the MATE cascade LoD to be 22.2 pM after a total assay time of 2 h. Putting these results into perspective, we witnessed that the novel MATE cascade improved the assay time by 12-fold when compared with our previous circuit design^25^ and the sensitivity by 37-fold when compared to a nonamplifying circuit. Our MATE assays are isothermally functional at a temperature range of at least 20 to 35 °C. Looking into the future, we envision that the sensitivity and assay time of MATE can further be improved by incorporating a two-factor amplification design, which combines MATE-based target recycling with the release of an amplification strand, similar to our previous work.

Our study demonstrates that declustering-based magnetic assays benefit substantially from engineering DNA circuits and integrating mismatch and toehold exchange concepts into the magnetic signal amplification cascade. To yield a fast MATE circuit, it is recommended to place the mismatch at the first base-pair of the spontaneously dissociating domain adjacent to the last base-pair between the invader and the substrate. In combination with keeping the spontaneous dissociation domain to six base-pairs, this ensures rapid opening of the allosteric toehold and efficient target recycling. The primary toehold (TH_T_) should be ideally one base-pair longer than the secondary toehold (TH_F_). To fine-tune desired and undesired reaction pathways for the desired objective of novel applications, the base-pair identities of the mismatch, its nearest neighbors, and GC content of the toehold domains may be altered, as more unstable base-pairs lead to faster kinetics but also a higher degree of unwanted leakage. The MATE diagnostics cascade advances magnetic-based assays toward their real-world POC application by offering highly sensitive and rapid assays in a nonenzymatic and isothermal fashion.

## Supplementary Material



## Notes

Experimental details as mentioned in the text including chemicals, DNA sequences, sample preparation protocols, assay protocols, oxDNA simulations, and characterization techniques; supporting ACS measurements (Figure S1); limited declustering for partial displacement of L_DSD_ in the absence of a mismatched bp (Figure S2); kinetic rates for different mismatch positions S*x* (Figure S3); declustering in the presence of a blocker strand (Figure S4); acceleration of declustering in the presence of Mg^2+^ (Figure S5); influence of TH_T_ length α on declustering kinetics (Figure S6); recycling is enhanced for longer TH_F_ domain (Figure S7); concentration driven recycling enhances MATE efficiency (Figure S8); leakage-based declustering for all mismatch positions *x* (Figure S9); NUPACK simulations of DNA complexes L_Sta_y + L_DSD_ + S*x* (Figure S10); increasing F does not linearly improve MATE kinetics (Figure S11); signal lag for increasing target concentrations (Figure S12); magnesium influences declustering more than sodium (Figure S13); DNA sequences used in this work (Table S1); sequence and free energy of DNA used in studies on mismatch positions (Table S2); fit parameters for the comparison of spontaneous dissociation of 6 bps in the presence of different mismatch positions *x* (Table S3); information on blocker strands B and B* (Table S4); DNA sequences used for CG variation in Figure 4 (Table S5) (PDF)
